# Supplementary material for: Content-rich biological network constructed by mining PubMed abstracts
Source: BMC Bioinformatics. 2004 Oct 8;5:147. doi: 10.1186/1471-2105-5-147 (PMC528731; doi:10.1186/1471-2105-5-147)
Supplement: Additional File 2 — The original results of the above study (non-essential files are deleted to keep the file size under the limit set by BMC bioinformatics). [file 1471-2105-5-147-S2.bz2 › chilibotAdditionalFile2/dip10/255ID7911228E1014/html/CDKN1A_PCNA.html]

 


 **CDKN1A** and **PCNA** 
  
Found 1649 abstracts in PubMed, retrieved 10.  
 

 What does Google say? 
 PDF only 
| .edu only 

---

**Interactive relationship** (e.g. stimulation, inhibition, etc)

**Inhibitory relationship**- Western blot analysis showed that both treatment with EGF alone and treatment with EGF after PH induce the expression of cyclins D1 and A and of p21 cip1  [ **CDKN1A** ] , but inhibites the expression of cyclin  [ **PCNA** ]  E and p27 kip1.  Ref: 12876382 Cell Physiol Biochem, 2003
- p21 Waf1  [ **CDKN1A** ]  Cip1  [ **CDKN1A** ]  exerts a key role in driving this G 2 exit both by inhibiting cyclin  [ **PCNA** ]  B1 Cdk1 and cyclin  [ **PCNA** ]  A Cdk1 2 complexes, which control G 2 M progression, and by blocking the phosphorylation of pRb family proteins.  Ref: 12881433 EMBO JEMBO J, 2003
**Neutral relationship**

**Non-interactive relationship** (e.g. studied together, co-existance, homology, etc.)

- AIMS To correlate the expression of a series of apoptotic and oncogene markers including p53, Bcl 2, BAX, Bcl XL, p21WAF, 1 CIP1  [ **CDKN1A** ] , cyclin  [ **PCNA** ]  D1, HER 2 neu in thymic epithelial tumours with histological type, stage and resectability and to determine whether the information on HER 2 neu would be valuable in identifying patients who are eligible for anti HER 2 neu treatment.  Ref: 12877732 Histopathology, 2003
- In 19 of these 63 patients, p53, p21 Waf1  [ **CDKN1A** ] , and proliferating cell nuclear antigen  [ **PCNA** ]   **PCNA**  were examined immunohistologically, and to quantify the risk of lymph node metastasis, computer analysis was performed on the basis of quantification theory II, in which pathological lymph node metastasis pN was the objective variable and high or low expression of each of the three markers was the predictive variable.  Ref: 12866574 J Exp Clin Cancer Res, 2003
- Both 5% human fecal water and 4 micro mol L selenite greatly increased the mRNA level of the cyclin  [ **PCNA** ]  dependent kinase inhibitor gene p21 waf1  [ **CDKN1A** ] .  Ref: 12888658 J Nutr, 2003
- Most thymomas and thymic carcinomas were negative for Bcl XL, p21WAF, 1 CIP1  [ **CDKN1A** ]  and cyclin  [ **PCNA** ]  D1.  Ref: 12877732 Histopathology, 2003
- These events were accompanied by down regulation of the Raf 1 MEK ERK pathway as well as diminished expression of Bcr Abl and cyclin  [ **PCNA** ]  D1, cleavage of p21 CIP1  [ **CDKN1A** ]  and pRb, and induction of the stress related kinases JNK and p38 MAPK.  Ref: 12893773 BloodBlood, 2003
- These deletions define a genomic region that includes cki 1 and cki 2, adjacent genes encoding members of the Cip  [ **CDKN1A** ]  Kip family of cyclin  [ **PCNA** ]  dependent kinase inhibitors.  Ref: 12885569 Dev Biol, 2003
- The cyclin  [ **PCNA** ]  dependent kinase inhibitor p21CIP WAF  [ **CDKN1A** ]  is a positive regulator of IGF 1 induced cell proliferation in MCF 7 human breast cancer cells.  Ref: 12867429 J Biol ChemJ Biol Chem, 2003
- Prediction of lymph node metastasis by p53, p21 Waf1  [ **CDKN1A** ] , and  **PCNA**  expression in esophageal cancer patients.  Ref: 12866574 J Exp Clin Cancer Res, 2003
- The aim of this study was to assess the effect of Fe chelators on p53 and the mechanism involved in the chelator mediated increase in mRNA levels of the universal cyclin  [ **PCNA** ]  dependent kinase inhibitor p21 CIP1  [ **CDKN1A** ]  WAF1  [ **CDKN1A** ]  Gao, J. and Richardson, D.R.  Ref: 12869419 CarcinogenesisCarcinogenesis, 2003
